# 

**Published:** 1904-07

**Authors:** 


### NOTICE.

In accordance with the provisions of Section 1423 of the Code of Georgia and of an amendment thereto, notice is hereby given that a competitive examination will be held in the city of Atlanta, on Thursday, July 14, 1904, at ten o'clock a. m., of all applicants for the position of assistant physician to the State Sanitarium at Milledgeville.

This examination is for the purpose of providing for any vacancy that may occur or for a possible increase of the medical staff.

For further information address

JAMES B. BAIRD, M.D.,
Atlanta,
or C. J. MONTGOMERY, M.D.,
Augusta.

---

DR. W. B. RUSH, of Miami, Fla., is taking a three months' vacation in the White mountains, New Hampshire.

---

DR. C. G. GIDDINGS and Dr. F. W. McRae are both taking their vacation in Europe.

***NOTICE.— This Journal and the International Journal of Surgery one year for $1.00 cash, to new subscribers. No out-of-town checks accepted unless 10c. is added for cost of cashing. Strictly to new subscribers.***

**This Journal and Atlanta Semi-weekly Journal one year for $1.25 cash in advance. No checks. Strictly to new subscribers.**

**This Journal and Atlanta Semi-weekly Journal one year for $1.25 cash in advance. No checks. Strictly to new subscribers.**

**NOTICE.—This JOURNAL and THE INTERNATIONAL JOURNAL OF SURGERY one year for $1.00 cash, to new subscribers. No out-of-town Checks accepted unless 10c. is added for cost of cashing.**

**This Journal and Atlanta Semi-weekly Journal one year for $1.25, cash in advance. No checks. Strictly to new subscribers.**

